# Artificial Intelligence Approaches for Prediction of Compressive Strength of Geopolymer Concrete

**DOI:** 10.3390/ma12060983

**Published:** 2019-03-25

**Authors:** Dong Van Dao, Hai-Bang Ly, Son Hoang Trinh, Tien-Thinh Le, Binh Thai Pham

**Affiliations:** University of Transport Technology, Hanoi 100000, Vietnam; sonth@utt.edu.vn (S.H.T.); tienthinhle.vn@gmail.com (T.-T.L.); binhpt@utt.edu.vn (B.T.P.)

**Keywords:** geopolymer concrete, compressive strength, adaptive network-based fuzzy inference system, sensitivity analysis

## Abstract

Geopolymer concrete (GPC) has been used as a partial replacement of Portland cement concrete (PCC) in various construction applications. In this paper, two artificial intelligence approaches, namely adaptive neuro fuzzy inference (ANFIS) and artificial neural network (ANN), were used to predict the compressive strength of GPC, where coarse and fine waste steel slag were used as aggregates. The prepared mixtures contained fly ash, sodium hydroxide in solid state, sodium silicate solution, coarse and fine steel slag aggregates as well as water, in which four variables (fly ash, sodium hydroxide, sodium silicate solution, and water) were used as input parameters for modeling. A total number of 210 samples were prepared with target-specified compressive strength at standard age of 28 days of 25, 35, and 45 MPa. Such values were obtained and used as targets for the two AI prediction tools. Evaluation of the model’s performance was achieved via criteria such as mean absolute error (MAE), root mean square error (RMSE), and coefficient of determination (*R*^2^). The results showed that both ANN and ANFIS models have strong potential for predicting the compressive strength of GPC but ANFIS (MAE = 1.655 MPa, RMSE = 2.265 MPa, and *R*^2^ = 0.879) is better than ANN (MAE = 1.989 MPa, RMSE = 2.423 MPa, and *R*^2^ = 0.851). Sensitivity analysis was then carried out, and it was found that reducing one input parameter could only make a small change to the prediction performance.

## 1. Introduction

Conventional Portland cement concrete, widely used in civil construction, has a significant impact on the greenhouse effect due to manufacturing processes of constituent materials, such as cement binder, coarse and fine aggregates, as well as reactions in the cement hydration process [1,2]. As reported in the work of Mo et al. [3], the cement industry released about 5% of the global CO_2_ emissions. Geopolymer concrete (GPC) then appeared as one of the most important inventions in the concrete industry for reducing carbon dioxide emissions in civil engineering activities. Geopolymer is produced from a geopolymerization process involving aluminosilicate materials such as fly ash, metakaolin, steel slag, together with an alkaline liquid activator, for instance, sodium hydroxide and/or sodium silicate [4]. The use of fly ash or steel slag in GPC manufacturing largely reduced CO_2_ emissions in comparison to cement, i.e., up to 5–6 times lower [5]. For that reason, this alternative cement-less material has now received great attention from researchers over the world for both environmental and economic considerations. GPC has been successfully applied in a number of structural construction applications such as beam, column, slab, tunnel lining, pavement, etc. The main reason relied on the fact that GPC could perform similar mechanical behavior as conventional Portland cement concrete [6,7,8,9,10,11,12].

However, to achieve the desired mechanical properties of GPC, researchers have to explore a mixture design and/or an appropriate selection of ingredients in laboratory conditions. For instance, Panda et al. [13] prepared five different mixes of GPC in order to find the most suitable proportion for utilization in 3D concrete printing. Those ingredients were listed as fly ash, ground granulated blast-furnace slag, silica fume, alkaline reagent, water, and river sand. Ferdous et al. [14] developed a multi-step methodology in order to identify the best mix proportions to obtain GPC with a given target of compressive strength at 28 days. Naghizaded et al. [15] experimentally developed a new mix-design method for fly ash-based geopolymer mortar in order to improve its compressive strength. Mixture design parameters that have been investigated in these studies can be stated as activator-to-fly ash ratio, sodium silicate-to-sodium hydroxide ratio, and molarity of sodium hydroxide. In order to evaluate the compressive strength degradation of GPC immersed in different chemical solutions, Albitar et al. [12] proposed two distinct mix proportions to obtain fly ash-based and granulated lead smelter slag-based GPC, respectively. GPC was activated by alkaline liquids of a combination of sodium silicate (Na_2_SiO_3_) and sodium hydroxide (NaOH). Further, the strength of GPC was highly dependent on the buffering capacity of the corresponding alkaline liquids [16]. It was also shown that the temperature had a significant influence in activating the aluminosilicate materials during the geopolymerization process [1]. In general, all of these laboratory experiments were complicated, and cost and time consuming. Despite all the efforts, it is not always possible to carry out a large number of ingredients and/or mixture proportion for manufacturing GPC. Moreover, experimental works might not have the required ability to investigate the relationship between various input parameters and the target mechanical properties of GPC, particularly in terms of compressive strength. It clearly showed that a more robust manner is thus required for better understanding and predicting the effective properties of GPC.

Generally, there are three approaches that are commonly used to predict compressive strength, namely computational modeling [17], parametric multi-variable regression model [18], or the artificial intelligence approach [19]. Out of these approaches, artificial intelligence (AI) approaches have been broadly used by researchers in the fields of civil engineering in the last two decades [20,21,22]. Such data-driven methods, mainly artificial neural network (ANN) and fuzzy logic (FL), have become popular because of their prediction ability in many engineering applications [23,24], especially in terms of mechanical strength of GPC [25,26,27,28,29,30,31]. As an example, Topçu et al. [32] developed both ANN and FL models to predict the 7-, 28-, and 90-day compressive strength of fly ash-based concrete. In this study, data of several input parameters such as water, sand, and fly ash contents, and the compressive strength of material as an output were collected from the literature. Such trained multilayer neural networks have exhibited a great potential application for predicting the compressive strength of concretes (minimum value of the coefficient of determination, *R*^2^, was 0.96). However, the relationship between material composition and mechanical strength was not investigated in this work. This is important in order to quantify the impact of input parameters on the effective properties of concrete. In another attempt, Prasad et al. [33] successfully constructed an ANN model to predict the 28-day compressive strength of high-volume fly ash-based concrete. Again, the ANN algorithm used in this study has not provided an evaluation between output results and input parameters such as fly ash/binder and water/powder ratios. It is worth noting that such information is relevant and could strongly influence the mechanical behavior of GPC [1,3]. Despite all the variation in weight of ingredients to form GPC, most of the developed ANN or FL prediction models are deterministic and no sensitivity analysis has been investigated.

As mentioned above, the literature review reveals that not many studies have pointed out the use of AI approaches to predict the compressive strength of GPC with additional steel slag as both coarse and fine aggregates. Although numerous experimental efforts have been carried out in the literature, there are still more robust computational methods needed to investigate the influence of such ingredient mix proportions to the final mechanical properties of GPC.

Consequently, the main objectives of this work can be stated as:

- To affirm the feasibility of using steel slag in GPC as both fine and coarse aggregates;
- To propose AI algorithms in order to predict the compressive strength of GPC using steel slags as aggregates;
- To perform the sensitivity analysis of input parameters on compressive strength of GPC.
  With that aim, the outline of the present study is the following:
- Preparation of test specimens: GPC samples were prepared using different mixtures of fly ash, steel slag as coarse and fine aggregates, activated by sodium hydroxide liquid, and sodium silicate solution;
- Mechanical tests: Compression testing procedure was then applied to investigate the 28-day compressive strength of GPC samples, all data were then collected;
- Development of AI models, such as adaptive network-based fuzzy inference system (ANFIS) and artificial neural networks (ANN), for predicting the compressive strength of GPC samples. The prediction performance of these models was evaluated by the coefficient of determination (*R*^2^) along with root mean square error (RMSE), mean absolute error (MAE);
- Sensitivity analysis: The impact of input variable fluctuations on the output results was quantified using the Monte Carlo approach.

## 2. Data Preparation and Testing

In this investigation, three specified compressive strength grades of geopolymer concrete (f’c = 25, 30, 35 MPa) as regards to the ACI 211-91 [34] were designed. To this aim, four constituent materials were used and named as simulation inputs X_1_, X_2_, X_3_, and X_4_ that correspond to the fly ash, sodium silicate solution, sodium hydroxide in solid state, and water contents, respectively. These constituents highly influence the final compressive strength of GPC, which is output Y for the prediction study.

Fly ash (Class F—ASTM C618-03 [35]) was purchased from Pha Lai Thermal Power Plant (Hai Duong, Vietnam). The main corresponding chemical compositions of fly ash, namely SiO_2_, Al_2_O_3_, Fe_2_O_3_, CaO, MgO, K_2_O, Na_2_O, TiO_2_, SO_3_, and loss on ignition (LOI) are measured as: 51.74, 24.53, 5.59, 0.81, 1.95, 4.42, 0.11, 0.76, 0.31, and 8.98 wt.%, respectively. The passing sieved volume of the latter are 95.00, 51.67, 33.06, and 16.77 wt.% and correspond to the sieved size range as 30, 20, 10, and 5 μm, respectively. Anhydrous NaOH (Purity = 98.5%; Density = 2.1 g/cm^3^) and the sodium silicate solution (Relative Density = 1.42; 39–40.03 wt.% of Na_2_SiO_3_) were purchased from Viet Tri Chemical Factory (Phu Tho, Vietnam). Dumped steel slag, which served as fine and coarse aggregates, was supplied by Thai Nguyen Iron and Steel Industrial Park (Thai Nguyen, Vietnam). Prior to the casting of GPC samples, steel slag aggregates were crushed into different particle sizes then prepared following the ASTMC136 [36] and mixed together according to the ASTMC33 [37]. Steel slag aggregates are separated into two groups: (1) 4.75–19 mm particle size, which served as coarse aggregates; and (2) 0.15–4.75 mm range size, acting as fine aggregates.

In order to correctly activate fly ash (X_1_), sodium hydroxide solutions with different molar mass (10, 12, and 14 M) were prepared by dissolving anhydrous NaOH powders (X_3_) in the corresponding amount of water (X_4_). Sodium silicate solution (X_2_) was then homogeneously mixed with the sodium hydroxide solution at a different mass ratio to obtain the final activated fly ash solutions.

The mixing process is carried out with a forced mixing concrete mixer with 60 L capacity (Daiwa Kenko Co., ltd., Tokyo, Japan). The mixture was kneaded following the ASTM C192 standard [38] with a dry mixing time of 3 min and a wet mix of 5 min. After mixing, the slump of the mixture was measured, then casted for the testing procedures. The mixture was added into the cylindrical mold and compacted vibrating for 90 s to obtain standard cylindrical samples with the ratio of radius and height of 150 mm × 300 mm. In order to avoid evaporation, the samples were covered by plastic wrap. After 48 h, samples were removed from molds and stored at ambient laboratory conditions.

At the age of 28 days, compressive strength of GPC samples was measured using a 2000 KN Universal testing machine YU-2000DIV (Tokyo Testing Machine Inc., Tokyo, Japan). The testing procedure was carefully carried out following the ASTM C39 [39]. The results of compressive strength of specimens are shown in Table 1. For 1 m^3^ of the mixture, the content of fly ash (X_1_) varied from 475.33 to 509.29 kg, Na_2_SiO_3_ (X_2_) varied from 135.81 to 178.25 kg, NaOH (X_3_) content ranged from 17.13 to 34.84 kg, and the water amount (X_4_) was taken from 28.53 to 52.58 kg. For all the samples, the amount of steel slag was kept constant at 2387 kg with the mass ratio between coarse and fine aggregates was 7:3 (1670 kg coarse and 716.1 kg fine aggregates). Moreover, the as-obtained values of corresponding compressive strengths (Y) are briefly detailed in Table 1. It should be highlighted that only destructive tests have been conducted in this study, even though alternative non-destructive experimental methods are also the subject of many other studies [40,41].

## 3. Methods Used

### 3.1. Artificial Neural Network (ANN)

**Figure 1:**
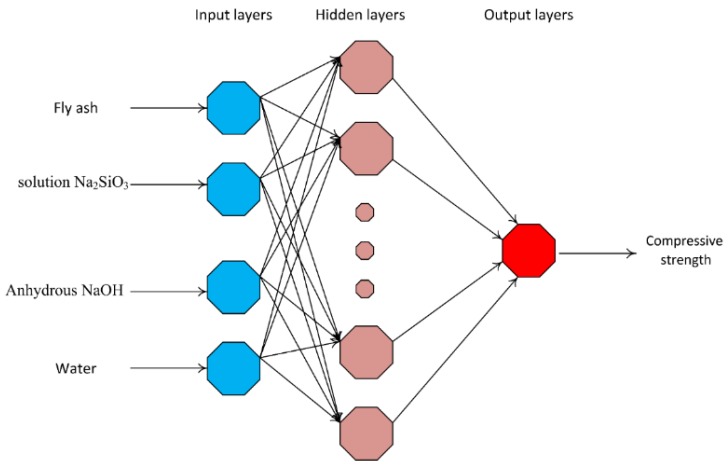
Architecture of the ANN model used in this study [47]. Reprinted with permission from [47]. 2012, Francisco J. de Cos Juez et al.

Artificial neural network (ANN) is one of the most widely used models in the field of machine learning and data mining. This model, inspired by the biological neural networks of human brains, is an interconnected system of nodes that could be trained to predict the desired output from the given input [42]. This technique exhibits significant advantages not found in traditional computational models as it does not require assumptions or predefined constraints about the form of the model (for instance between dependent and independent variables) [43]. It has ability to detect and analyze complex nonlinear relationships in data itself [44]. This method can also provide the pattern during training (generalization) [43]. Most importantly, due to its parallel processing capability, the ANN is powerful in large data problems [45]. There are three basic components that concept an ANN model architecture, named input layers, hidden layers, and output layers [21] (Figure 1). In this study, the ANN model containing five hidden layers was selected to perform intermediary computations for mapping the inputs and the outputs.

**Figure 2:**
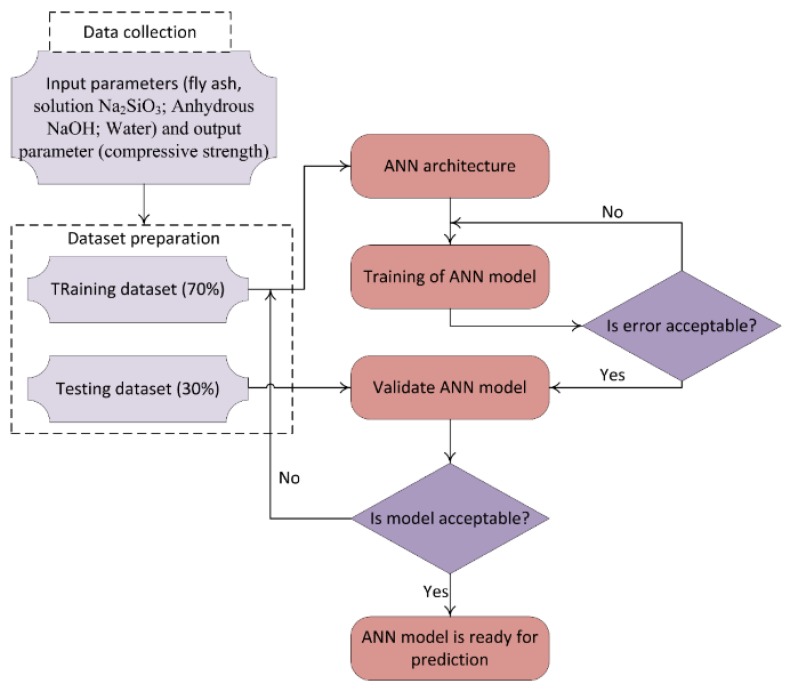
Methodology of development of an efficient artificial neural network (ANN) model [48]. Reprinted with permission from [48]. 2013, Mohd Idris Shah Ismail et al.

In the ANN, the sigmoid function was also used to compute the weights of the relationship between inputs and outputs:

(1) $$y=f(x)=\frac{1}{1+e^{-x}}$$

where *x* = (*x*_1_, *x*_2_, … *x_k_*) are the inputs and *y* is the output (compressive strength of GPC samples) [46]. In this study, the ANN was used to predict compressive strength of geopolymer concrete through several main steps (Figure 2). Firstly, the data collected were divided into two parts with a ratio of 7:3, where 70% of data was used for generating the training dataset and 30% remaining data was used to generate the testing dataset. Secondly, a training dataset was used to build the ANN model (5 hidden layers and based on Scaled Conjugate Gradient method). Thirdly, the predicted results were compared to experimental data using different criteria such as mean absolute error (MAE), root mean square error (RMSE), and the coefficient of determination (*R*^2^) to validate and test the efficiency of the proposed ANN model.

### 3.2. Adaptive Neuro Fuzzy Inference System (ANFIS)

**Figure 3:**
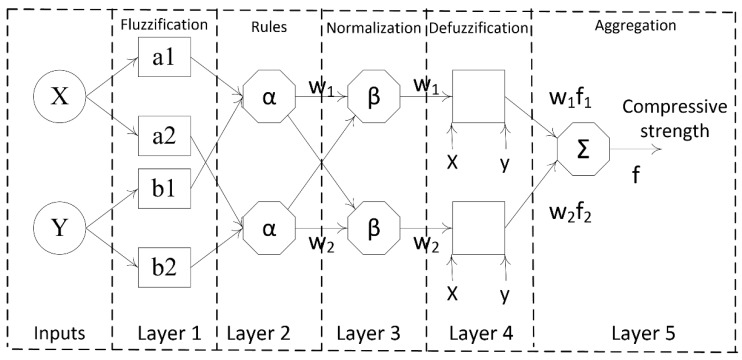
The structure of the adaptive neuro fuzzy inference (ANFIS) algorithm [53]. Reprinted with permission from [53]. 2016, Ziqiang Bi et al.

Adaptive neuro fuzzy inference system (ANFIS) is a popular artificial intelligence technique which is a combination of artificial neural networks and fuzzy logic [49]. The main task of artificial neural networks is to optimize the membership capacities for reducing the mistake rate in the output whereas the main task of fuzzy logic rules is to simulate expert knowledge [50]. In the ANFIS, fuzzy logic rules are used as fuzzy if-then rules with reasonable membership functions to create the stipulated input-output sets [51]. An ANFIS includes five layers [52] which are represented by some nodes and node functions (Figure 3).

Layer 1: It is called the fuzzification layer which includes the defined membership functions of the input parameters, and every node of this layer acts as a membership function. In this layer, Gaussian membership function is used to calculate the output, which is defined as a degree of membership value, expressed as follow:

(2) $$\mu_{Ui}\left(x\right)=\exp\left[-\frac{\left(x-a_{i}\right)}{2\varepsilon_{i}^{2}}\right]$$

where *a_i_* and ε*_i_* are defined as parameters of a membership function.

Layer 2: Every node of this layer is used to send the product out by multiplying the incoming signals. The main aim of this layer is to execute the fuzzy AND of the previous parts of the fuzzy rules by the following equation:

(3) $$w_{i}=\mu_{Ui}\left(x\right)*\mu_{Vi}\left(y\right)$$

where *U_i_* and *V_i_* are input layers; *x* and *y* are inputs of node *i*.

Layer 3: It is called as the normalized layer which is used to normalize the membership functions. Node *i^th^* of this third layer calculates the ratio of the *i^th^* firing strength rules to the sum of all firing strength rules as follows:

(4) $${\bar{w}}_{i}=\frac{w_{i}}{\sum_{i}w_{i}}.$$

Layer 4: It is called as the defuzzification layer which executes the consequent part of the fuzzy rules. Every node of this defuzzification layer is a square node with a node function as follows:

(5) $${\bar{w}}_{i}f_{i}=w_{i}*\left(m_{i}x+n_{i}y+r_{i}\right)$$

where *m_i_*, *n_i_*, and *r_i_* are defined as linear parameters.

Layer 5: It is called as the combination layer or the output of fuzzy system which is calculated by summing up the outputs of previous layers as following equation:

(6) $$\sum_{i}\bar{w}f_{i}=\frac{\sum_{i}w_{i}f_{i}}{\sum_{i}w_{i}}.$$

**Figure 4:**
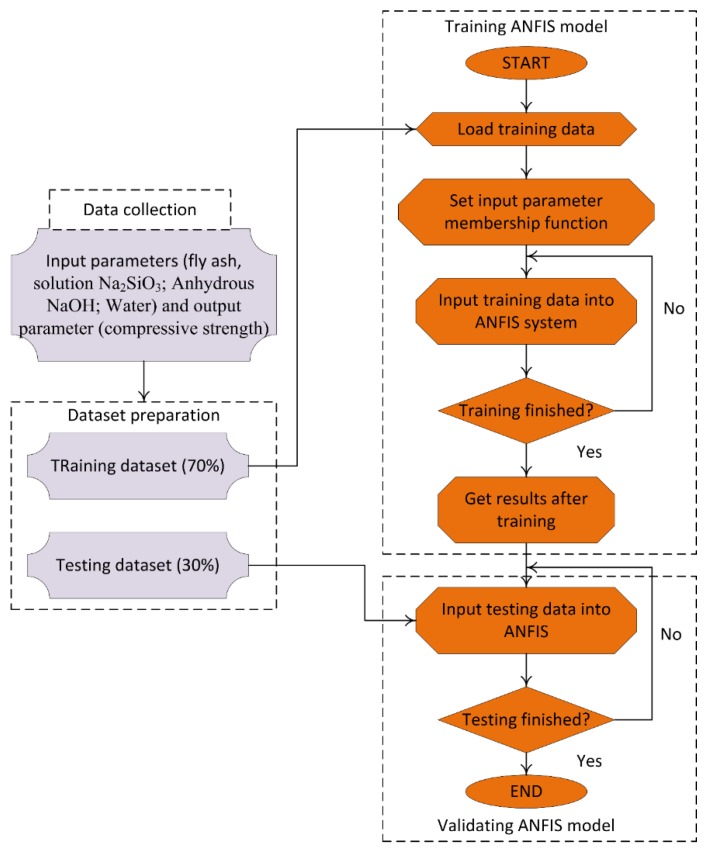
Methodology of development of an efficient ANFIS model [54]. Reprinted with permission from [54]. 2013, Hue-Yu Wang et al.

In this study, the development of the ANFIS for prediction of the compressive strength of geopolymer concrete was carried out in several steps: (i) Preparation of the datasets for modeling, in which the same datasets generated for the development of the ANN were used for development of the ANFIS; (ii) training the model, in which the training dataset was used to train the ANFIS for construction of the model (ANFIS-based subtractive clustering algorithm); and (iii) validating the model, in which the testing dataset, which was used in the training process, was used to validate the model using several criteria such as RMSE, MAE, and *R*^2^ (Figure 4).

### 3.3. Validation Methods

To validate the performance of the models, various validation methods were used in this study, namely root mean square error (RMSE), mean absolute error (MAE), and the coefficient of determination (*R*^2^). These measures are well-known techniques for quantifying the accuracy of the artificial intelligence models [55]. Out of these methods, the mean squared difference between outputs and targets infers RMSE, the mean magnitude of the errors infers MAE, and *R*^2^ represents the correlation between targets and outputs [46]. Basically, lower RMSE and MAE indicate better accuracy of the model. In contrast, higher *R*^2^ (ranged from 0 to 1) indicates better accuracy of the model [55]. These measures can be calculated by the following equations [55]:

(7) $$\mathrm{MAE}=\frac{1}{n}\sum_{i=1}^{n}\left(u_{i}-{\overset{⌢}{u}}_{i}\right)$$

(8) $$\mathrm{RMSE}\;=\;\sqrt{\frac{1}{n}\sum_{i=1}^{n}{\left(u_{i}-{\overset{⌢}{u}}_{i}\right)}^{2}}$$

(9) $$R^{2}=1-\frac{\sum_{i=1}^{n}{\left(u_{i}-{\overset{⌢}{u}}_{i}\right)}^{2}}{\sum_{i=1}^{n}{\left(u_{i}-\overset{⌢}{u}\right)}^{2}}$$

where *u_i_* is defined as the actual output, ${\overset{⌢}{u}}_{i}$ is the predicted output, $\overset{⌢}{u}$ is defined as the mean of the *u_i_*, and *n* is the number of the samples.

## 4. Results and Discussion

This investigation aims at performing both a laboratory experiment as well as a prediction of GPC compressive strength entirely benefiting from steel slag as fine and coarse aggregates. This could be an attempt to facilitate further investigations in this field and give an overview of the choice of aggregates and corresponding mix proportion for GPC. Detailed results for compressive strength (Y) are given in Table 1, along with all the corresponding ingredients (X_1_, X_2_, X_3_, and X_4_) used as input parameters for the AI algorithms. For concrete composite materials, the input parameters are usually set as ratios, for instance, water/powder, water/binder, water/cement, coarse or fine aggregate/powder, etc. However, in this study, it is successfully demonstrated that a simple form of input parameters can also be used. X_1_ as fly ash (wt.%), X_2_ as Na_2_SiO_3_ solution (wt.%), X_3_ refers to NaOH solid form (wt.%), and X_4_ as water (wt.%) were selected as input variables for the AI prediction tools. The corresponding compressive strength of samples was used as prediction target (Y).

### 4.1. Prediction Capability of ANN and ANFIS

**Figure 5:**
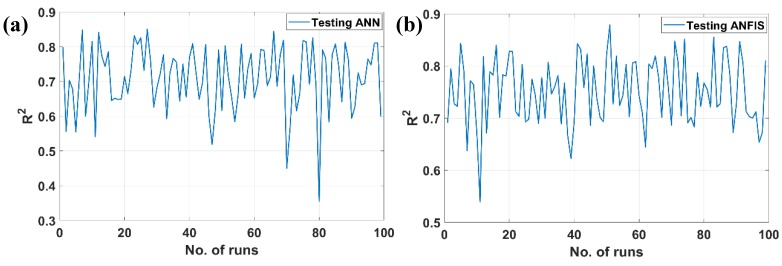
Error distribution (*R*^2^) of different models for 100 runs: (**a**) testing ANN; (**b**) testing ANFIS.

The first objective of this research was to compare the performance of the two artificial intelligence methods, namely ANN and ANFIS, based on results obtained by mechanical tests. The coefficient of determination (*R*^2^) was chosen as a principal measurement for the precision of prediction models in this study. The prediction results were evaluated by the goodness of fit between predicted and target values. The closer the values of *R*^2^ were to 1, the better of the fit regression models proposed by AI algorithms. Regarding to the prediction capability, the goodness of fit models in testing part of the data was selected as the main criterion to compare the performance between ANN and ANFIS algorithms. Figure 5 shows the *R*^2^ error for testing ANN and testing ANFIS algorithms for 100 different simulations. As for clarification, 70% of the as-obtained experimental data was randomly taken to construct the AI prediction tools, thus the corresponding *R*^2^ values were different for each simulation (Figure 5). The best performance, represented by the highest value of *R*^2^, of ANN method was observed at realization 27 with *R*^2^ = 0.851, whereas that of the ANFIS algorithm was at run 51 with *R*^2^ = 0.879. The mean values of *R*^2^ for 100 runs were *R*^2^ = 0.711 and *R*^2^ = 0.751 for ANN and ANFIS methods, respectively. The corresponding values of MAE and RMSE were MAE = 1.655 and RMSE = 2.265 for the ANFIS model, and MAE = 1.989 and RMSE = 2.423 for the ANN model, respectively. Such results could demonstrate that the ANN and ANFIS algorithms possessed the ability to well-predict compressive strength of GPC. Moreover, the ANFIS outperformed the ANN method regarding the best performance, as well as for *R*^2^ mean values.

**Figure 6:**
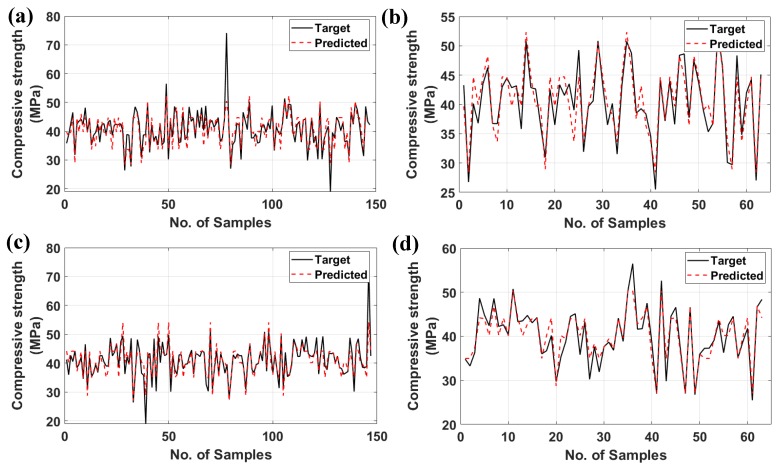
Target and predicted values of compressive strength of different models: (**a**) training ANN, (**b**) testing ANN, (**c**) training ANFIS, and (**d**) testing ANFIS.

Figure 6 shows the as-obtained experimental data (black, continuous line) and the predicted values (red, discontinuous) from the training and the testing data of the ANN along with the ANFIS algorithms. It is clearly indicated that the predicted compressive strength of both algorithms was in strong coherence with the target ones. Such an assumption is confirmed with the training part (70% of data or 147 samples) as well as the validation/testing one (30% of data or 63 samples) for both ANN and ANFIS algorithms.

**Figure 7:**
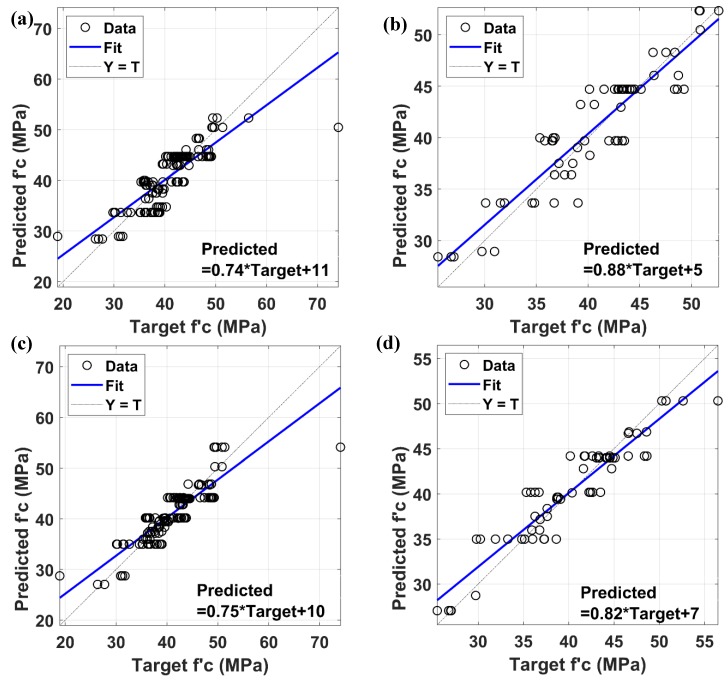
Correlation results analysis of ANN and ANFIS models: (**a**) training ANN, (**b**) testing ANN, (**c**) training ANFIS, and (**d**) testing ANFIS.

The linear fit line and its equations are given in Figure 7 for the training and testing of ANN and ANFIS models. The performance in predicting the compressive strength values of the training and testing data were satisfactory with *R*^2^ = 0.693 and *R*^2^ = 0.851 for the ANN algorithm, and *R*^2^ = 0.752 and *R*^2^ = 0.879 for the ANFIS algorithm, respectively. Again, compressive strength values of GPC containing steel slag as both fine and coarse aggregates can be successfully predicted by ANN and ANFIS without attempting too many experiments as well as reasonable errors. Such observations have shown that these two algorithms are practicable methods for predicting compressive strength values of steel slag aggregates GPC. As ANFIS applied neural networks to optimize its algorithm, it is natural that in this case its performance is better than ANN.

### 4.2. Sensitivity Analysis

In many situations, the input parameters are perhaps not trivial, and the larger the number of inputs, the more complicated the prediction task. Therefore, the sensitivity analysis of input parameters is necessary in order to reduce the number of input parameters. The next section is dedicated to study the influence of input parameters (X_1_, X_2_, X_3_, and X_4_) to the final prediction target (Y) by doing simulation in excluding successively each of them. The number of input parameters for the prediction numerical tools was chosen as 3 and the value of the excluded one is set to 0. A total number of eight groups of simulation were then carried out by excluding successively X_1_ (fly ash), X_2_ (Na_2_SiO_3_), X_3_ (NaOH), and X_4_ (water), running with the ANN and ANFIS algorithms, respectively. The performance of both methods on the prediction of compressive strength is chosen as the coefficient of determination (*R*^2^), showing the regression fit between experimental and predicted results. The best prediction performance for each case is represented by the maximum value of *R*^2^ between outputs and targets in testing data.

**Figure 8:**
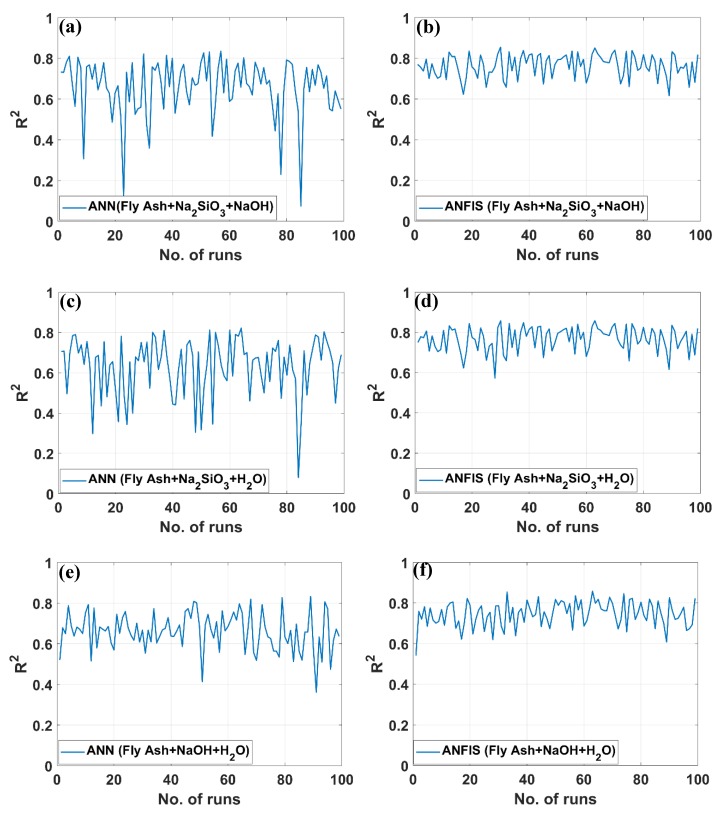

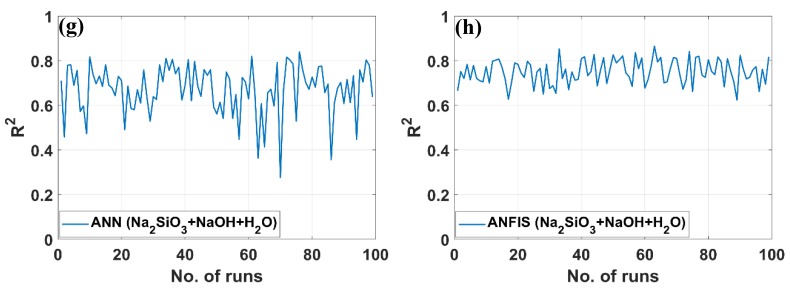
Error distribution (*R*^2^) of ANN and ANFIS models using three input parameters for 100 different runs: (**a**) testing ANN of Fly Ash + Na_2_SiO_3_ + NaOH, (**b**) testing ANFIS of Fly Ash + Na_2_SiO_3_ + NaOH, (**c**) testing ANN of Fly Ash + Na_2_SiO_3_ + H_2_O, (**d**) testing ANFIS of Fly Ash + Na_2_SiO_3_ + H_2_O, (**e**) testing ANN of Fly Ash + NaOH + H_2_O, (**f**) testing ANFIS of Fly Ash + NaOH + H_2_O, (**g**) testing ANN of Na_2_SiO_3_ + NaOH + H_2_O, and (**h**) testing ANFIS of Na_2_SiO_3_ + NaOH + H_2_O.

Figure 8 demonstrates the *R*^2^ values of 800 simulations using the ANN algorithm and the ANFIS algorithm. Results are plotted in Figure 8a–h for simulations in excluding X_4_ (H_2_O), X_3_ (NaOH), X_2_ (Na_2_SiO_3_), and X_1_ (fly ash). The *R*^2^ values of best performance were equal to *R*^2^ = 0.835, 0.822, 0.833, and 0.839 for the ANN method, and *R*^2^ = 0.855, 0.858, 0.858, and 0.864 for ANFIS, with simulations without H_2_O, NaOH, Na_2_SiO_3_, and fly ash, respectively. It is noteworthy that the *R*^2^ values of ordinary simulations (four input parameters) using the ANN and ANFIS methods are *R*^2^ = 0.851, 0.879, respectively. It is observed that the prediction effectiveness reduced in both cases but rather in an accepted range of about *R*^2^ = 0.02. It implies that compressive strength of GPC can be predicted with only three input parameters within a reasonable error. In all cases, the ANN is less effective than ANFIS algorithm regarding the difference between *R*^2^ values (about *R*^2^ = 0.02). Last but not least, it is monitored that for 400 simulations, ANN algorithm possessed many runs that the *R*^2^ values are in the range of *R*^2^ = 0.09–0.58, while the ANFIS method gave higher values of *R*^2^, i.e., *R*^2^ = 0.58–0.86. Again, it can be concluded that ANFIS algorithm is more stable and more effective in predicting compressive strength of GPC.

**Figure 9:**
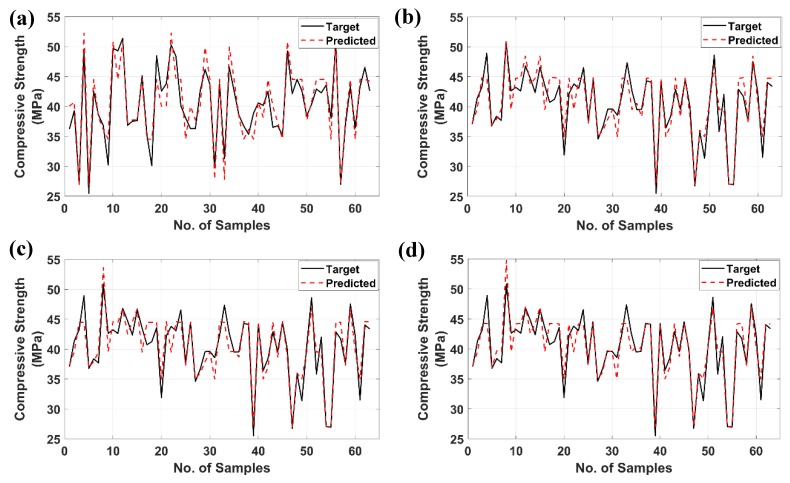
Target and ANFIS-predicted values of compressive strength using three input parameters: (**a**) Testing Fly Ash + Na_2_SiO_3_ + NaOH, (**b**) testing Fly Ash + Na_2_SiO_3_ + H_2_O, (**c**) testing Fly Ash + NaOH + H_2_O, and (**d**) testing Na_2_SiO_3_ + NaOH + H_2_O.

Figure 9 compares predicted and experimental compressive strength using the ANFIS algorithm. Simulation results for four combinations of input parameters are plotted: Fly ash, Na_2_SiO_3_, NaOH (Figure 9a); Fly ash, Na_2_SiO_3_, H_2_O (Figure 9b); Fly ash, NaOH, H_2_O (Figure 9c); and Na_2_SiO_3_, NaOH, H_2_O (Figure 9d). These comparisons correspond to best performance realizations for testing data, based on maximum *R*^2^ values of 100 runs. For conclusion, compressive strength of GPC using the steel slag as aggregates can be well predicted, even the number of input parameters decreased from four to three.

**Figure 10:**
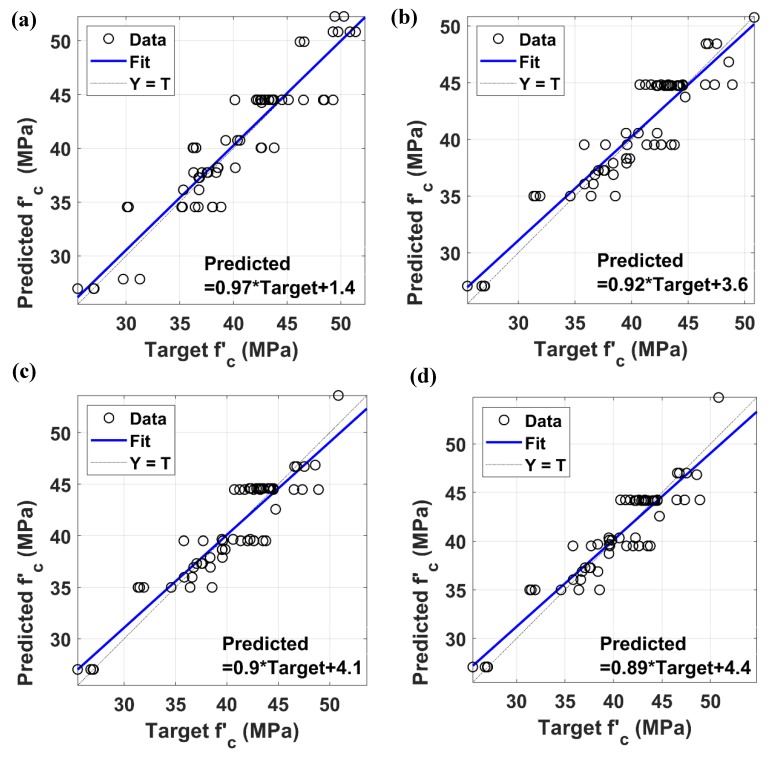
Correlation results analysis of ANFIS model using three input parameters: (**a**) Testing Fly Ash + Na_2_SiO_3_ + NaOH, (**b**) testing Fly Ash + Na_2_SiO_3_ + H_2_O, (**c**) testing Fly Ash + NaOH + H_2_O, and (**d**) testing Na_2_SiO_3_ + NaOH + H_2_O.

The linear fit lines along with its equations and *R*^2^ values are plotted in Figure 10, representing the best performance runs regarding testing data using ANFIS model. *R*^2^ values obtained in the three cases are similar, i.e., *R*^2^ = 0.858, 0.858, and 0.855 while running simulations in excluding Na_2_SiO_3_, NaOH, and H_2_O, respectively. Besides, applying zero values to fly ash input parameter gave a slightly better *R*^2^ value, i.e., *R*^2^ = 0.864. Thus, it can be deduced that reducing one input parameter in the combination of input variables is acceptable due to a small variation between *R*^2^ values, compared to the full simulation case with four inputs (i.e., *R*^2^ = 0.879). Certainly, the presence of fly ash, Na_2_SiO_3_, NaOH, and H_2_O, as well as their content ranges, are crucial for the compressive strength of GPC. These ingredients chemically interact with each other and the final as-obtained product has significant influence on GPC compressive strength. The influence of these parameters are well known and can be found in the literature, such as, the importance of silicate solution (Na_2_SiO_3_ or K_2_SiO_3_) in alkaline solutions (NaOH or KOH) [56] or the molar concentration of the alkaline solution [57,58]. However, regarding the sensitivity analysis of these input parameters, it was found that excluding one variable has no influence to the performance of AI tools. Prediction of compressive strength could be achieved using only three input parameters with reasonable error range.

All of the statistical values summarized in Table 2 demonstrate that the proposed ANN and ANFIS models are suitable and predict the compressive strength values close to experimental values. It is also noticed that ANFIS appears to be a better candidate for the prediction of compressive strength of GPC than ANN. Even in case of excluding one input parameter, ANFIS outperformed ANN as compared by *R*^2^ values. Moreover, regarding the average values of *R*^2^ for 100 simulations, ANN algorithm presented an unstable method in contrast to ANFIS. As presented above, this is due to the high fluctuation of *R*^2^ values simulated by ANN—a lot of runs contained low *R*^2^ values, whereas the minimum values of *R*^2^ for ANFIS was about *R*^2^ = 0.6 (see Figure 7). Moreover, RMSE and MAE values are also presented in Table 2. It was found that these values were in excellence agreement with the observation obtained with *R*^2^ error. In all cases, RMSE and MAE values of ANFIS were smaller than that of ANN, showing that ANFIS outperforms ANN.

Throughout the errors calculated by both ANN and ANFIS methods, it could be easily concluded that the approach by using ANFIS-optimized algorithms are very promising numerical tools for the prediction of compressive strength of GPC with entirely steel slag aggregates. As there is no official standard for the mix-design of GPC until this moment, such tool could provide useful information that might be useful for engineers to save time, costs and increase work effectiveness.

## 5. Conclusions

Geopolymer concrete is an eco-environmental material which can be used as a replacement cement concrete in construction. The compressive strength of GPC is an important parameter which reflects its quality. In this study, a quantitative investigation of the influence of four input variables, such as: fly ash, Na_2_SiO_3_, NaOH, and H_2_O on the effective compressive strength of GPC was carried out. In order to better understand the complex relationship between inputs and output of such engineering problem, two “black box” based on AI numerical models (ANN and ANFIS) were constructed in order to: (i) Predict the compressive strength of GPC in function of four input parameters and (ii) propagate the uncertainties of inputs on output results using Monte Carlo simulation. Based on a total number of 210 data arising from laboratory experiments, ANN and ANFIS models were constructed, trained, and validated to predict the compressive strength of GPC. The MAE, RMSE, and the coefficient of determination (*R*^2^) were used to evaluate the prediction performance of the two proposed algorithms. Such numerical tools could be useful for engineers in order to reduce time, cost, and laboratory experiments while manufacturing GPC. A total number of 800 numerical Monte Carlo simulations were performed next in order to quantify the effect of each input fluctuations to the prediction output. The uncertainty quantification was also useful to test the robustness of the proposed prediction models under uncertainties of input parameters. In conclusion, the two proposed methods performed well for prediction of the compressive strength of GPC but the ANFIS (MAE = 1.655, RMSE = 2.265, and *R*^2^ = 0.879) outperformed the ANN (MAE = 1.989, RMSE = 2.423, and *R*^2^ = 0.851). It is noteworthy that the quantity of steel slag aggregates was kept constant for all the samples in this investigation. In addition, we used only one type of fly ash and did not consider the variability of this component for the modeling. Thus, these input parameters might be considered in future study, so that a better understanding for this mix-design issue might be achieved. Besides, other important mechanical properties of the GPC such as fracture strength and durability should be investigated. As prospective works, we also plan to study the prediction capability of other AI algorithms for comparison purposes, among which the supported vector machine (SVM) or optimized ANFIS would be potential candidates.

## Figures and Tables

**Table 1:** Mix proportion of geopolymer concrete (GPC) using steel slag aggregates.

Samples	Fly Ash X_1_ (kg)	Na_2_SiO_3_ X_2_ (kg)	NaOH X_3_ (kg)	H_2_O X_4_ (kg)	Compressive Strength Y (MPa)
**GPC 01**	475.33	135.81	26.64	52.58	29.73
**GPC 02**	475.33	178.25	19.98	39.44	25.48
**GPC 03**	509.29	135.81	22.83	45.07	38.84
**–**	–	–	–	–	–
**–**	–	–	–	–	–
**GPC 208**	491.72	158.05	24.64	38.58	44.52
**GPC 209**	482.41	172.94	22.47	35.18	38.03
**GPC 210**	509.29	135.81	29.86	38.04	50.74
**Minimum**	475.33	135.81	17.13	28.53	18.92
**Maximum**	509.29	178.25	34.84	52.58	74.12
SD *	10.82	13.87	2.99	4.46	6.24

* SD: Standard deviation.

**Table 2:** Error values of ANN and ANFIS models.

Validation Criteria	Case	Best Performance		Average Values	
		ANN	ANFIS	ANN	ANFIS
***R*^2^**	Fly Ash + Na_2_SiO_3_ + NaOH + H_2_O	0.851	0.879	0.711	0.751
	Fly Ash + Na_2_SiO_3_ + NaOH	0.835	0.855	0.657	0.763
	Fly Ash + Na_2_SiO_3_ + H_2_O	0.822	0.858	0.630	0.767
	Fly Ash + NaOH + H_2_O	0.833	0.858	0.653	0.746
	Na_2_SiO_3_ + NaOH + H_2_O	0.839	0.864	0.670	0.750
**RMSE**	Fly Ash + Na_2_SiO_3_ + NaOH + H_2_O	2.423	2.265	3.309	3.180
	Fly Ash + Na_2_SiO_3_ + NaOH	2.370	2.115	3.559	3.024
	Fly Ash + Na_2_SiO_3_ + H_2_O	2.456	2.057	3.745	2.985
	Fly Ash + NaOH + H_2_O	2.406	2.045	3.668	3.113
	Na_2_SiO_3_ + NaOH + H_2_O	2.231	1.999	3.555	3.091
**MAE**	Fly Ash + Na_2_SiO_3_ +NaOH + H_2_O	1.989	1.655	2.449	2.135
	Fly Ash + Na_2_SiO_3_ + NaOH	1.835	1.614	2.661	2.166
	Fly Ash + Na_2_SiO_3_ + H_2_O	1.923	1.550	2.746	2.141
	Fly Ash + NaOH + H_2_O	1.888	1.603	2.676	2.200
	Na_2_SiO_3_ + NaOH + H_2_O	1.721	1.504	2.595	2.161
